# Transcriptome profile analysis of two *Vicia faba* cultivars with contrasting salinity tolerance during seed germination

**DOI:** 10.1038/s41598-020-64288-7

**Published:** 2020-04-29

**Authors:** Fangwen Yang, Hongwei Chen, Changyan Liu, Li Li, Liangjun Liu, Xuesong Han, Zhenghuang Wan, Aihua Sha

**Affiliations:** 1 Hubei Collaborative Innovation Center for Grain Industry/Engineering Research Center of Ecology and Agricultural Use of Wetland of Ministry of Education, Yangtze University, Jingzhou, P.R. China; 2 Institute of Food Crops, Hubei Academy of Agricultural Sciences/Hubei Key Laboratory of Food Crop Germplasm and Genetic, Wuhan, P.R. China

**Keywords:** Biological techniques, Plant sciences

## Abstract

Faba bean (*Vicia faba* L.) is an important food legume crop. Salinity soils severely constrain the production of faba bean, however, the seed germination of faba bean, which is a vital plant growth stage, is sensitive to salinity. Planting improved varieties of faba bean, which exhibit salt tolerance in seed germination stage, is an optimal strategy for faba bean product. To investigate the genes dynamics during the seed germination stage under salinity, RNA-seq method was used to investigate genome-wide transcription profiles of two faba bean varieties with contrast salt-tolerance during the seed germination. A total of 4,486 differentially expressed genes (DEGs) were identified among the comparison of salt-tolerant variety Y134 and salt-sensitive variety Y078 treated with salinity or not. Of these, 1,410 candidate DEGs were identified as salt-stress response genes. Furthermore, 623 DEGs were identified as variety-specific response gene during seed germination at 16 h or 24 h with salt treatment. Based on the pathway enrichment according to the Kyoto Encyclopedia of Genes and Genomes database (KEGG), these DEGs involving in cell wall loosening (e.g., xyloglucan endotransglucosylase/hydrolase, chitinase, and expansin), hormone metabolism (e.g., LEA genes, genes associated with ABA or ethylene signal pathway), chromatin remodeling (e.g., chromatin structure proteins, LHP1), small interfering RNA pathway, etc., were significantly up-regulated in salt-tolerance variety with salt treatment, indicating that they play critical roles in regulation of seed germination. The results indicated that a clearer mechanism of gene regulation that regulates the seed germination responding to salinity in faba bean. These findings are helpful to increase the understanding of the salt tolerance mechanism of crops during seed germination, and provide valuable genetic resource for the breeding of salt-tolerant faba bean varieties in future.

## Introduction

Salt stress is one major production constraint factor of crops. In word-wide, more than 800 million (ha) of land was affected by salinization, and it is increasing due to erroneous cultivation practices^1^. Salinity strongly affects plant with the aspects of growth, yield, grain quality and composition^2^. Salt stress engages in osmotic stress, the imbalance of ions, as well as secondary stresses, for instance, the imbalance of nutrition and the stress of glycophyte oxidation^3^. Salt stress can changes gene expression to affect cell wall properties indirectly, whereas the chemical properties of cell wall components can be changed by the directly physical interaction of Na^+^ with them^4^. Chemically, the Na^+^ in the apoplast can accumulated to a high level due to soil salinity. As a result, the interactions of Na^+^ and negatively charged sites were increased within cell wall polymers, and the apoplastic pH was also influenced. In plants, the salt stress finally affects the modulation of metabolism, the expression of gene, and the activity of protein. Accordingly, the composition of cell wall, the processes of transport, the size and shape of cell, and the architecture of root are changed.

Faba bean (*Vicia faba* L.) is cultivated in more than 60 countries as cool season food legume crop with the third most importance^5^. China is one of the main countries growing faba bean, followed by Ethiopia, Morocco, and Australia. Faba bean provides proteins for human consumption as well as serving as a prime livestock feed^6^. Salinity soils severely constrained the product of faba bean in semi-arid regions^7^. Faba bean is sensitive to salinity stress that resulted in 20–50% yield loss^2^. Salinity threats germination of seed, plant growth, rhizobium symbiosis, development of root nodule, and nitrogen-fixed capacity in legumes^8^. Among the plant growth cycles, seed germination is a critical stage that promises plant survival and reproduction. Thus, selective breeding and planting varieties with tolerant salinity during seed germinating is crucial for faba bean production.

Seed germination means that the quiescent, dry seed start to uptake water and the embryonic axis elongate^9^. It is determined by the interaction of the increasing embryo growth potential and the decreasing covering layer resistance^10^. Many elements correlated to cell wall loosening play roles in the processes, such as expansins, xyloglucan endotransglycolases/hydrolases, endo-(1,4)-β-D-glucanases, chitinase, and apoplastic reactive oxygen species. The wall-loosening enzymes regulate cell turgor, which drives cell enlargement according to the cell wall extensibility, to create the driving forces for water uptake by growing cells^11,12^. In recent years, a lots of studies have been reported concerning the molecular basis of seed germination, including identification of salt tolerance related molecular markers, quantitative trait loci (QTL) mapping of salt tolerance during germination. For example, 10 genomic regions were correlated with tolerance to salinity in the germination and seedling stage in *Arabidopsis*^13^. One SNP (single nucleotide polymorphisms) was identified to significantly linked to salt tolerance during germinating, and 5 candidate genes were responsive to soybean salt salinity^14,15^. In cowpea, 3 SNPs were highly correlated to tolerance of salinity at the stage of germination^16^.

The genome of Faba bean is over 13,000 Mb with over 85% of repetitive DNA, which make it difficult to assemble the whole genome with the sequencing technologies. It is also difficult to develop the maps of genetics and physics, the cloning based on map in faba bean^17,18^. High-throughput approaches such as transcriptome analysis are important tools to discover the molecular basis concerning abiotic stresses in plant. In legumes species such as chickpea, soybean and *Medicago truncatula*, a handful of studies have been carried out for abiotic stress response through transcriptome analysis^19–21^. In this study, to investigate genes involved in regulation of seed germination in response to salinity in faba bean, two varieties contrastingly tolerant to salinity were used to carry out transcriptome analysis by means of RNA-seq technology. Many differentially expressed genes (DEGs) were discovered between the two varieties under salinity conditions during seed germination, which help us clarifying the key genes in regulation of seed germination under salinity in faba bean. These findings could facilitate for awareness of the genetic mechanism involved in salt tolerance during seed germinating in crops, and provide reference for the salt-tolerant breeding of faba bean varieties.

## Results

### Morphological changes

**Figure 1:**
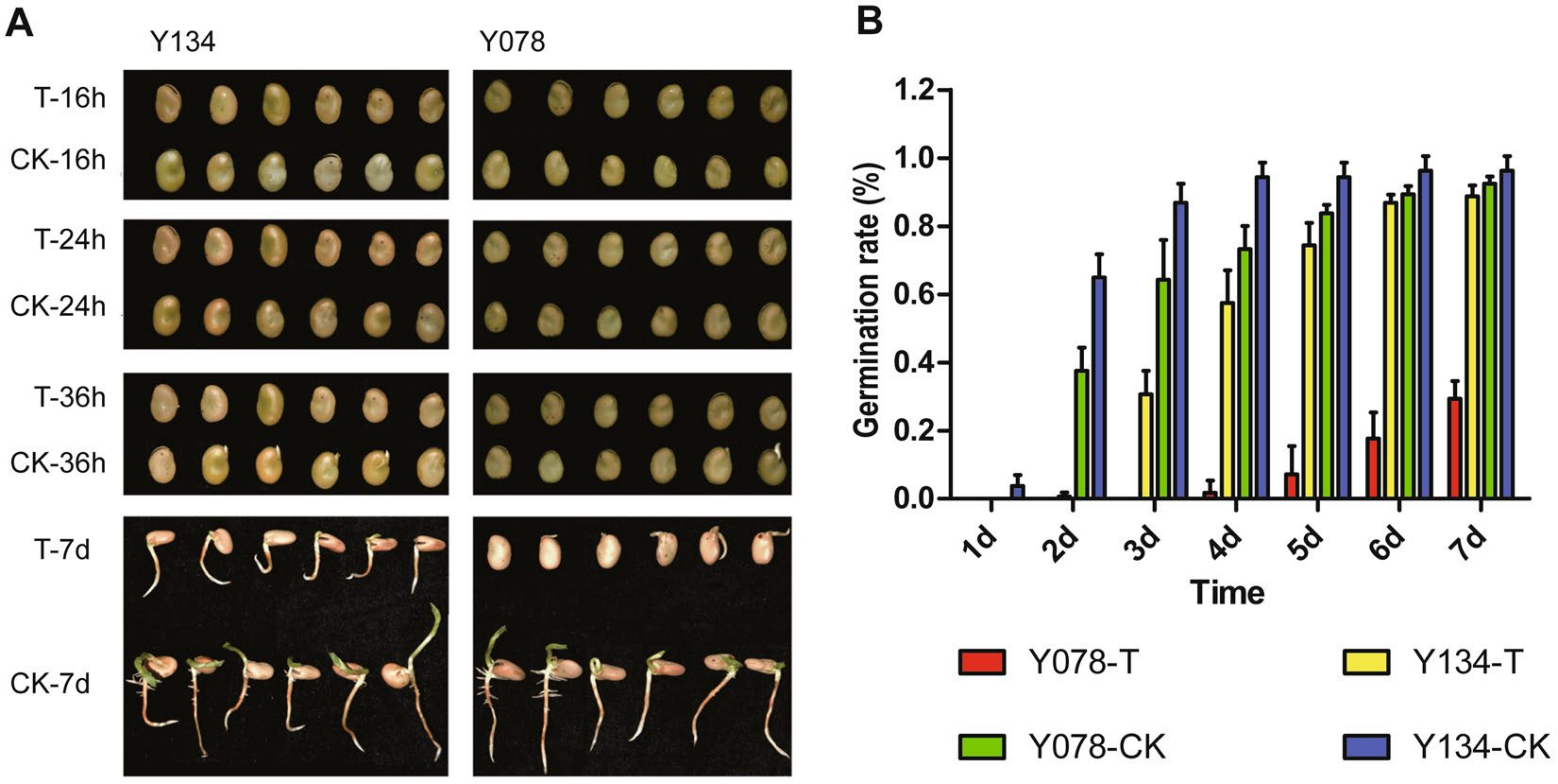
The morphology and germination rate of faba bean seeds. (**A**) The morphology of germinating seeds of faba bean varieties Y134 and Y078. T-16h, T-24h, T-36h, and T-7d represent that seeds were photographed at 16 h, 24 h, 36 h, and 7 d treated with salinity, respectively. CK-16h, CK-24h, CK-36h, and CK-7d represent that seeds were photographed at 16 h, 24 h, 36 h, and 7 d treated with water, respectively. (**B**) Statistics of seed germinate rate. Y078-T, Y134-T indicate that varieties Y134 and Y078 were treated with salinity; Y078-CK, Y134-CK represent that varieties Y134 and Y078 were treated with water.

In our previous study^22^, we have screened out one salt tolerant (Y134) and one salt sensitive (Y078) variety during the seed germinating from 67 faba bean landraces. It showed that Y134 and Y078 were highly tolerant and sensitive to salt stress, respectively. To further investigate the molecular base of seed germinating in response to salt, we also characterized the germinating process of Y134 and Y078. More than 50% and 30% of Y134 and Y078 seeds began to germinate under control condition (Fig. 1A). However, the seed germination of Y134 were 0.6% and 30.6% under salinity stress at 2 d and 3 d, respectively, whereas the seeds of Y078 under salinity stress were not germinated at 2 d and 3 d (Fig. 1B). It could be inferred that the superiority of genetic regulation related to salt tolerance played important roles in germination of Y134 at germinating stage. Thus, we selected seeds of Y134 and Y078 during the germinating point at 16 h and 24 h under salt treatment and control condition to investigate the genome-wide transcription profiles.

### Quality control, *de novo* assembly and annotation

**Figure 2:**
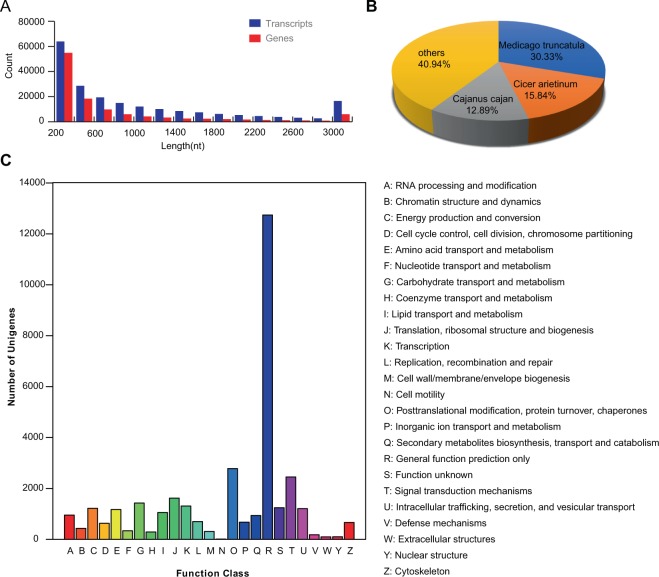
Assembled transcripts assessment. (**A**) The length distribution of assembled Unigenes statistics. (**B**) The proportion of homologous species annotating with Nr annotation based on the transcripts. (**C**) KOG function classification of *Vicia_faba*_L transcripts.

The germinating seeds of Y134 and Y078 treated with salinity or not at 16 h and 24 h were collected for preparation of the RNA samples. Eight samples (i.e., T1–16, CK1–16, T1–24, CK1–24, T2–16, CK2–16, T2–24, and CK2–24) were collected, and each sample had three biological replicates. T1, T2 represents Y134 and Y078 under salt treatment, and CK1, CK2 represent the control groups of Y134, Y078, respectively. −16 and −24 represent the time points at 16 h and 24 h, respectively. Finally, a total of 24 libraries were constructed and used for sequencing. A total of 218 million raw reads were generated. After quality control, the clean reads were remained for subsequent analyses. The statistics of clean reads for each library is shown in Table S1. A total of 116,093 unigenes and 207,506 transcripts were obtained after *de novo* assembly. The maximum length of the assembled unigenes was 19, 795 nt, whereas the mean length of unigenes was 850 nt and N50 (index meaning that 50% of the sequence contained in contigs, or scaffolds, is equal to or greater than this length) was 1964 nt (Table S2). The distribution of sequence length for unigenes and transcripts are showed in Fig. 2A.

The unigenes were annotated using BLASTX with Nr, Swiss-Prot, KEGG, and COG/KOG (E-value < 1e-5), in which 54,696, 16,354, 34,810, and 31,974 unigenes were annotated, respectively. The main three species of species distribution are *Medicago truncatula* (30.33%), *Cicer arietinum* (15.84%), *Cajanus cajan* (12.89%), which belong to leguminous plants. The unigenes of Vicia faba showed high homologous with the species *Medicago truncatula*, *Cicer arietinum*, *Cajanus cajan*, *Glycine max*, *Trifolium subterraneum*, *Pisum sativum*, *Glycine soja* with the counts ranging from 1484 to 16, 591 (Fig. 2B). KOG function classification of the unigenes was shown in Fig. 2C. The largest category is the general function prediction, followed by the categories of post-translational modification, protein turnover, chaperon, and signal transduction mechanism. The clean reads of 24 libraries were mapped to the assembled unigenes and the mapping ratios ranged from 40.87% to 85.13% (Table S3). The gene expression levels are shown in Table S4. Sequence saturation analysis showed that the above reads in each library were sufficient to approach saturation (Fig. S1).

### Gene expression profiles in germinating seeds of faba bean

**Figure 3:**
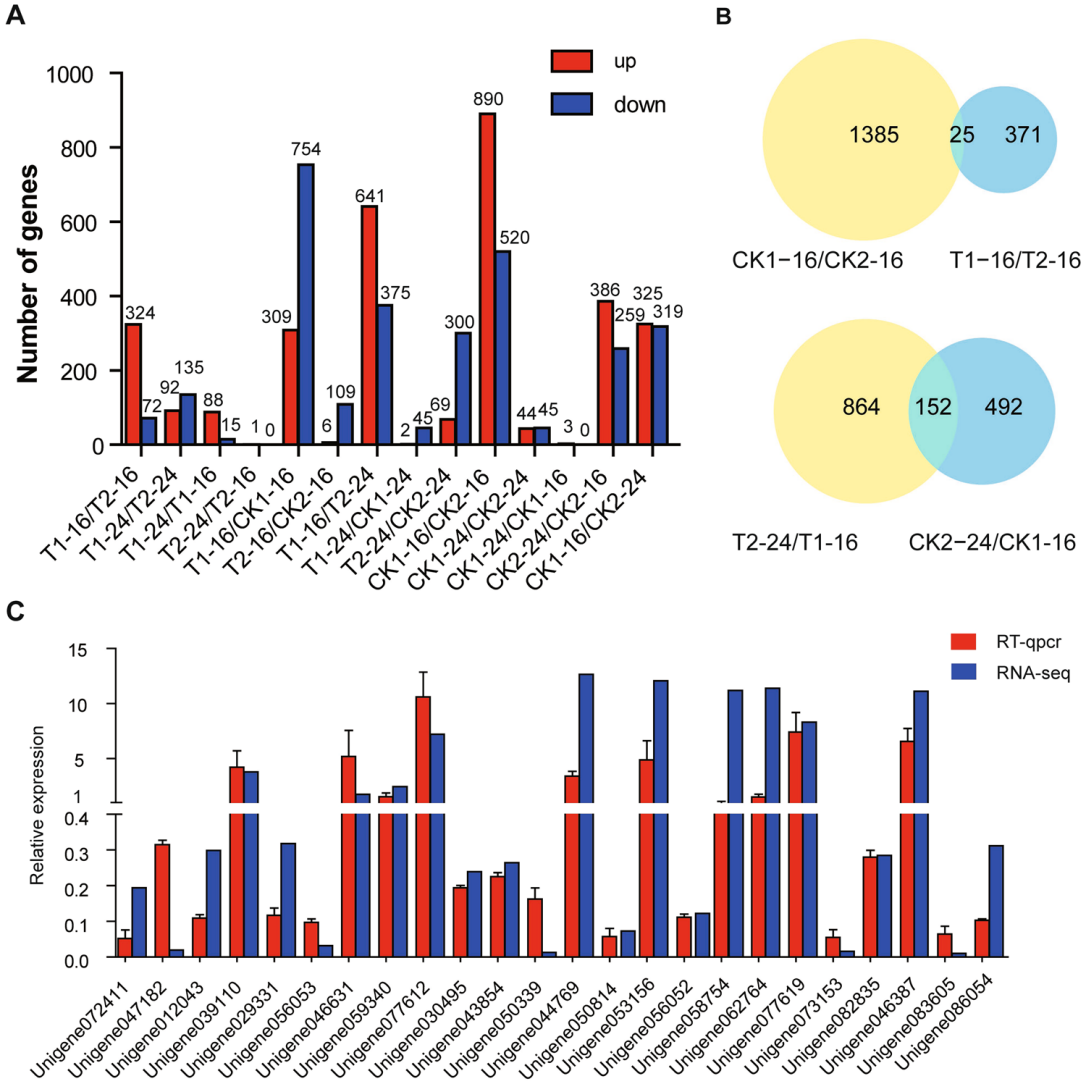
Differentially expressed genes among the groups and Quantitative RT-PCR validation. (**A**) Differentially expressed genes (DEGs) in two faba bean varieties treated with salt or control. T1–16, T1–24, CK1–16, and CK1–24 indicate variety Y134 treated with salt or control at 16 h, 24 h, respectively; T2–16, T2–24, CK2–16, and CK2–24 refer to as variety Y078 treated with salt or control at 16 h, 24 h, respectively. (**B**) Venn diagram showing the number of DEGs. (**C**) Validation of the expression patterns of DEGs by RT-qPCR. The housekeeping gene NADH dehydrogenase subunit 4 (NADHD4) was used as an internal reference gene.

A total of 4,486 DEGs were identified in at least one pairwise comparison (Fig. 3A, Table S5). To investigate genes associated with variety-specific tolerance of salinity, the DEGs identified in the comparison between T1–16 and T2–16, T1–24 and T2–24 were focused on. A total of 396 DEGs were identified between T1–16 and T2–16 (324 up-regulated and 72 down-regulated), and 227 DEGs were identified between T1–24 and T2–24 (92 up-regulated and 135 down-regulated). More DEGs were identified in the comparison of 16 h than that of 24 h, indicating that more genes were differentially expressed in the two varieties in earlier stage of seed germination under salinity treatments. In control groups, 1410 DEGs (890 up-regulated and 520 down-regulated) were identified between CK1–16 and CK2–16, and 89 DEGs (44 up-regulated and 45 down-regulated) were identified between CK1–24 and CK2–24, in which only 13 and 4 DEGs also existed in the comparison of T1–16 vs. T2–16, T1–24 vs. T2–24, respectively. The results suggested that the remarkable change in gene expression might be responsive for the different salinity tolerance of Y134 and Y078.

In order to investigate whether the delayed expression of genes affect the tolerance to salinity in seed germination stage, the pairwise comparisons of T1–16 vs. T2–24 and CK1–16 vs. CK2–24 were conducted. A total of 641 DEGs were up-regulated and 375 DEGs were down-regulated in the comparison of T1–16/ T2–24 (Fig. 3A, Table S6), whereas 325 DEGs were up-regulated and 319 DEGs were down-regulated in the comparison of CK1–16 vs. CK2–24. There were 152 DEGs in common between T1–16 vs. T2–24 and CK1–16 vs. CK2–24 groups (Fig. 3B), which might be variety-specific DEGs regardless of salinity response. So the remained 864 DEGs that were only in T1–16 vs. T2–24 might be the delayed expressing genes in salt-sensitive variety (Fig. 3B, Table S6), in which 588 were up-regulated and 276 were down-regulated.

To investigate the genes related to regulation of seed germinating process, a total of 103 DEGs were detected in Y134 between 16 h and 24 h under salinity treatments, with 88 up-regulated and 15 down-regulated genes, only 17 up-regulated DEGs were identified between 16 h and 24 h under control conditions. Notably, no DEG was detected in both two comparisons, inferring that the gene dynamics were temporally induced by salinity in Y134. Intriguingly, only one DEGs were up-regulated in Y078 between 16 h and 24 h under salinity treatments, although 386 and 259 DEGs were up-regulated and down-regulated between 16 h and 24 h under control condition. It was suggested that many genes were temporally induced during germinating process under normal conditions in Y078, whereas few genes were induced under salinity condition.

Candidate genes related to responding to salinity stress in different varieties were also investigated. There was 1,063 DEGs (309 up-regulated and 754 down-regulated) and 47 DEGs (2 up-regulated and 45 down-regulated) in Y134 at 16 h and 24 h between salinity treatment and CK, respectively, whereas 119 DEGs (6 up-regulated and 113 down-regulated) and 369 (69 up-regulated and 300 down-regulated) DEGs were identified in Y078 between salinity treatment and CK at 16 h and 24 h, respectively. More DEGs were identified at 16 h and less DEGs at 24 h in Y134 compared to those in Y078, suggesting that genes were induced more rapidly in Y134 than Y078, which might be correlated to their different salinity tolerance (Fig. 3B).

### Gene ontology (GO) and KEGG enrichment analysis

GO assignments were used to classify the functions of DEGs identified in different pairwise comparisons. Comparatively, 17,993 out of the 116,090 reference genes were assigned with GO terms and served as a background for enrichment analysis. The GO annotation of 1,262 DEGs was shown in Table S7. In this study, we mainly focused on the DEGs existed in comparisons of two varieties treated by salinity at 16 h and 24 h. In the category of biological process, glycerophospholipid metabolic process, glycerolipid metabolic process, and phospholipid metabolic process were the most enriched items in the comparison of T1–16 vs. T2–16 (*P* < 0.05), whereas cofactor metabolic and biosynthetic process, coenzyme biosynthetic and metabolic process were significantly enriched in the comparison of T1–24 vs. T2–24 (*P* < 0.05) (Table S8). In the category of molecular function, transferase activity and transferring glycosyl groups were the most enriched items in the comparison of T1–16 vs. T2–16 (*P* < 0.05), whereas transferase activity, transferring alkyl or aryl (other than methyl) groups, and exonuclease activity were most enriched in the comparison of T1–16 vs. T2–24 (*P* < 0.05). In cellular component, macromolecular complex was most enriched in comparison of T1–16 vs. T2–16 and lipid particle were the most enriched item in the comparison of T1–24 vs. T2–24 (*P* <0.05). At the same time, some stress -related activities items such as transcription factor complex, nuclear transcription factor complex, RNA polymerase II transcription factor complex were significantly enriched in both comparisons (Table S8).

In addition, the functions of candidate DEGs in the salt-tolerant varieties at 16 hours and 24 hours (T1–16 vs. CK1–16, T1–24 vs. CK2–24) was investigated. In the category of biological process, metabolic process, single-organism process, single-organism metabolic process were the most enriched items in comparison of T1–16 vs. CK1–16 (*P* < 0.05), whereas there were most enriched in polysaccharide metabolic process and carbohydrate metabolic process in comparison of T1–24 vs. CK1–24 (*P* < 0.05) (Table S8). In the category of molecular function, transferase activity and transferring glycosyl were most enriched in comparison of T1–16 vs. CK1–16 and binding and catalytic activity were the most enriched items in comparison of T1–24 vs. T2–24 (*P* < 0.05). In the Cellular Component, macromolecular complex were the most enriched item in comparison of T1–16 vs. CK1–16 (*P* < 0.05), whereas cell part, cell, membrane and membrane part were most enriched in comparison of T1–24/CK1–24 (*P* < 0.05). Meanwhile, some stress-related activities items such as transcription factor complex, nuclear transcription factor complex, RNA polymerase II transcription factor complex were significantly enriched in both comparisons. (Table S8).

Likewise, KEGG pathway enrichment analysis was performed. There were 49 and 62 DEGs mapped to 45 and 49 KEGG pathways in the T1–16 vs. T2–16 and T1–24 vs. T2–24 comparison, respectively. The most enriched items were plant hormone signal transduction in T1–16 vs. T2–16, whereas protein processing in endoplasmic reticulum were most enriched in T1–24 vs. T2–24. It is noteworthy that 3 and 6 pathways were significantly (*P* ≤ 0.05) enriched by DEGs in the former and the latter comparison, respectively, and the pathway regulation of autophagy existed in both comparisons (Table S9).

**Figure 4:**
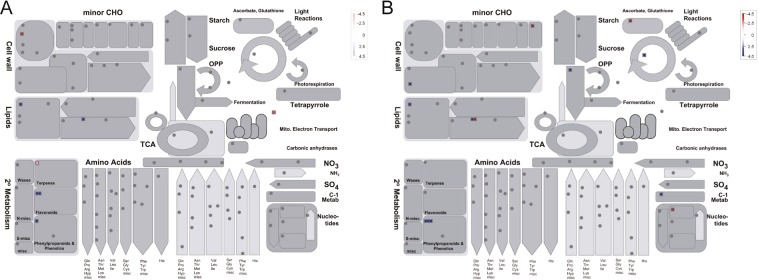
A metabolic pathway with a differential expression profile in T1–16/T2–16 and T1–16/T2–24. Compared to CK1 in 16 h of seed development. The differentially expressed genes (fold change > 2, q value < 0.05) between T1–16 vs. T2–16 and T1–16 vs. T2–24 were loaded into Mapman to generate an overview. On the log2 scale, dark blue and deep red represent higher and lower expression in T1–16 compared to T2–16 (**A**) and T2–24 (**B**), respectively.

In addition, 333 and 13 DEGs were assigned into 102 and 20 KEGG pathways in the T1–16 vs. CK1–16 and T1–24 vs. CK1–24 comparison, respectively. The number of the annotated pathways varies largely, and the DEGs are enriched in pathway of Cysteine and methionine metabolism, protein processing in endoplasmic reticulum, pentose and glucuronate interconversions in the comparison of T1–24 vs. CK1–24. The number of T1–16 vs. CK1–16 is much more than T1–24 vs. CK1–24. The path map drawn by Mapman to see the effect of salt stress on the gene expression of faba bean seeds. The results of Mapman mapping with the DEGs from T1–16 vs. T2–16 (Fig. 4A) and T1–16 vs. T2–24 (Fig. 4B) showed that the differential genes are mainly annotated in cell wall, lipids, secondary metabolism such as flavonoids, terpenes. Meanwhile, mitochondrial electron transport was only detected in T1–16/T2–16, but TCA cycle, nucleotides and ascorbate, glutathione were only detected in T1–16 vs. T2–24.

### Positive regulation of cell well loosening and hormone metabolism under salt stress

To investigate the genetic regulation of seed germination in response to salinity in faba bean, the transcriptomic profiles were investigated on the germinating seeds of the salinity-tolerant variety Y134 and the salinity-sensitive variety Y078 in this study. A large number of DEGs were identified between Y134 and Y078 under salinity at the time point of 16 h and 24 h, respectively. These candidate genes may play vital roles in variety-specific tolerance to salinity for faba bean. Overall, compared to Y087 variety, Y134 revealed robust gene dynamics when treated with salt at 16 h and 24 h time points.

**Figure 5:**
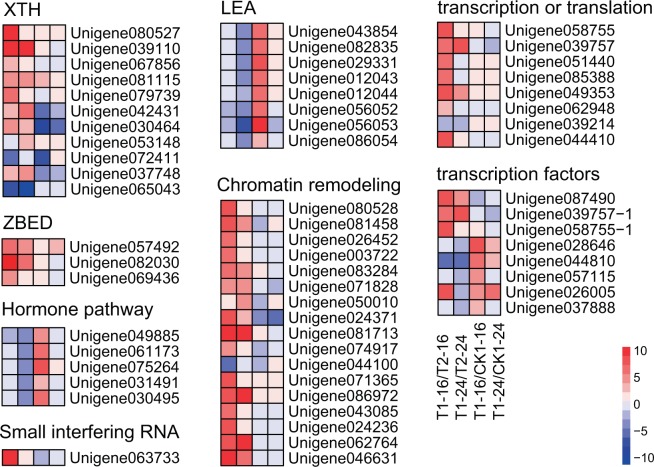
Heatmap of the gene expression profiles of 8 pathways in four pairwise comparisons, T1–16 vs. T2–16, T1–24 vs. T2–24, T1–16 vs. CK1–16 and T1–24 vs. CK1–24, respectively.

Some DEGs related to the regulation of cell wall loosening were identified Table S5, such as xyloglucan endotransglucosylase/hydrolase, chitinase, and expansin. Most of them were up-regulated in Y134 with the exception of hydrolase (Unigene072411) and expansin (Unigene065043), suggesting that the positive regulation of cell well loosening in Y134 was more sensitive than that in salinity-sensitive variety Y078, which is in favor of the plant cell expanding and removing the covering layer of seed to germinate (Fig. 5). However, xyloglucan endotransglucosylase/hydrolase family protein, Chitinase and extensin were down-regulated in salinity-tolerant variety Y134 (T1–16 group) treated with salt stress compared to control group, which indicated that salt stress inhibited seed germination by inhibiting cell wall expansion. This means that these genes that affect cell wall expansion during seed germination are likely to be key genes that affect seed germination under salt stress.

The down-regulation of genes related to hormone metabolism were also observed in salt stress treated group of salinity-tolerant variety Y134 compared to salinity-tolerant variety Y078. Five genes that involved in ABA or ethylene signal pathway were down regulated in Y134 at 24 h, including abscisic acid 8′-hydroxylase 2 (Unigene049885), phosphatase 2 C (Unigene075264, Unigene061173), AFP1 (Unigene031491), and ethylene-responsive transcription factor (Unigene030495), respectively. In addition, 8 LEA genes were also down-regulated in Y134 at 24 h under salt stress. However, these LEA genes were significantly up-regulated under salt treatment at 16 h compared with control group in Y134. LEA is the landmark gene of seed maturation, and it was regulated by the ABA signal pathway genes, such as ABI3, ABI4, ABI5 and DOG1^23^. It could be suggested that salt treatment possibly induced the higher expression levels of ABA and ethylene signal genes in salt sensitive varieties, furthermore suppressed the germination of seeds. On the contrary, the expression levels of ABA and ethylene signal genes in salt tolerant variety (Y134 variety) were kept relatively lower, inferring that the down-regulation of ABA and ethylene signal pathway genes in Y134 might promote the germination of seeds.

### Quantitative RT-PCR (qRT-PCR) analysis

RT-qPCR was conducted to validate the expression patterns of DEGs derived from the RNA-seq data. Twenty-four unigenes were selected from the list of DEGs based on their potential functions, such as hydrolase (Unigene039110, Unigene072411), 1-phosphatidylinosito l-3-phosphate 5-kinase (Unigene059340), autophagy-related protein (Unigene046631, Unigene044769), calcium-binding EF-hand protein (Unigene077612, Unigene077619), zinc ion-binding protein (Unigene047182), late embryogenesis abundant protein (Unigene012043, Unigene029331, Unigene056053, Unigene043854, Unigene056052, Unigene082835, Unigene086054) (Table S10). RT-qPCR results of the selected DEGs were well consistent with the RNA-Seq data, except for E3 ubiquitin-protein ligase UPL3-like and CCAAT-binding transcription factor. It implies that the expression levels calculated from RNA-Seq data was consistent with those generated from RT-qPCR, indicating that the results in this study are reliable although there was little discrepancy between RNA-seq and RT-qPCR (Fig. 3C).

## Discussion

Salinity affects plant growth and development with different aspects as one popular environmental stress factor on. Salt stress restricts water absorption, inhibits the growth of embryo axis, and leads to secondary seed dormancy^2^. Germination of seed is critical that the quiescent, dry seed start to uptake water and the embryonic axis elongate^9^. It is determined by the interaction of the increasing embryo growth potential and the decreasing covering layer resistance^10^. The mechanism of down-regulated seed germination under salt stress in faba bean is not well defined. In this study, The salt-tolerant variety (Y134) and the salt-sensitive variety (Y078) were compared to investigate the differences in genetic responses, which is expected to be correlated to salinity resistance. Based on the MAPMAN analysis, we found that the genes related to secondary metabolites including terpenes and flavonoids were significantly upregulated in salt-tolerant variety (Y134). It has demonstrated that flavonoids and terpenes play important regulatory role in plant growth, stress resistance and secondary metabolism. We inferred that these metabolites positively mediate in adapting the salt stress for faba bean seeds.

In plants, high intracellular Na^+^ and Cl^−^ concentrations restrains germination and causes seed death by limiting cell metabolism at the stage of dividing and expanding^11^. During the seed imbibition that uptake water, the osmotic potential and ion toxicity will be reduced if the concentrations of Na^+^ and Cl^−^ ions are excessive, which highly disturb germination process and decrease the germination of seed under sanlinity^12^. In the past years, the roles of cell wall loosening salt tolerance of plant have been reported for several crop plants such as rice^24^, maize^25^, barley^26^. Notably, in this study, we found that many genes associated with cell wall loosening, such as xyloglucan endotransglucosylase/hydrolase, chitinase and expansin, were up-regulated in the salt-tolerance variety Y134 under salt treatment (Fig. 5). For instance, expansin is a known cell-wall-loosening protein, which disrupts the hydrogen bonds connected the xyloglucan and cellulose microfibrils. The expression of expansin in different plant species is up-regulated by environmental clues of drought, salt, and heat shock. Previous studies have showed that salinity induced the transcript change of β-expansin in Sorghum bicolor and in the salt-resistant maize cultivar^23,27,28^. These up-regulated genes that identified in salt-tolerant faba bean variety may play critical roles in adaptation of salt stress and provide more genetic information on the future breeding of salt-resistant varieties.

In addition, other key factors regulates seed germination such as hormone metabolism, chromatin remodeling resulted from histone ubiquitination, methylation and acetylation, and epigenetic changes trigged by siRNA (small interfering RNA) and/or lncRNA (long non-coding RNA)^29^. Seed germination and dormancy are regulated by hormonal, which are highly conservative in seed plants. In this study, we found that several genes related to hormone metabolism were also down-regulated in salt-tolerant variety faba bean. Five genes (i.e., abscisic acid 8’-hydroxylase 2, phosphatase 2 C, AFP1, and ethylene-responsive transcription factor) that participated in abscisic acid (ABA) or ethylene signal pathway were down regulated in Y134 at 24 h. In addition, 8 LEA genes were down-regulated in Y134 at 24 h under salt treatment (Fig. 5). However, these LEA genes of Y134 were significantly up-regulated under salt treatment at 16 h compared with control group. LEA is the landmark gene of seed maturation, and it was regulated by the ABA signal pathway genes ABI3, ABI4, ABI5 and DOG1^30^. It has been documented that the suppression of ABA and ethylene pathway can promote germination^29^. The down-regulation of ABA and ethylene signal pathway genes in Y134 might promote the germination of seeds.

Meanwhile, those DEGs involved in regulation of chromatin remodeling, transcript or translation, may play important roles in seed germinating regulation of faba bean. Some genes involved in chromatin remodeling were up-regulated in Y134 under T1–16 vs. T2–16 and T1–24 vs. T2–24, such as chromatin structure proteins HMG1/2-like protein, Heterochromatin Protein 1-like (LHP1), AT hook motif DNA-binding family protein, methylation and acetylation proteins lysine-specific demethylase. The genetic regulation of seed germination has been clarified by Nonogaki^29^. The ubiquitination, methylation and acetylation of histone could cause elongation of transcription or silencing of gene, which mayregulate the dormancy and germination of seed. In addition, siRNA/lncRNA probably regulateseed germination by trigger the epigenetic changes. Previous study also showed that methylated metabolite could accumulate as osmopootectants and radical scavengers in salinity-tolerant species of^31^. Notably, the gene encoding protein argonaute 4-like (Unigene063733) was significantly up-regulated in Y134 at 16 h compared with control group. AGO proteins are component of the RNA-induced silencing complex (RISC) to participate in genesilencing. AGO4 is correlated to RdDM-mediated transcriptional gene silencing (TGS)^32^, and it positively regulates seed germination^33^. The argonaute 4-like protein are up-regulated in Y134, inferring that the salt tolerance of Y134 are involved in TGS regulation. In salt tolerant variety (Y134), the upregulation of genes related to chromatin remodeling, transcript or translation was absent in the control group (CK1–16) compared with treatment (T1–16), inferring that the superiority of Y134 for tolerance of salt stress rely on upregulation of these genes, to a certain extent.

Some other DEGs were also identified such as transcription factors CCHC-type Zinc finger (Unigene087490), CCAAT-binding transcription factor (Unigene039757), nuclear transcription factor Y subunit C8 (Unigene058755), heat shock factor protein (Unigene028646, Unigene044810), implying that these genes might be engaged in regulating the seed germination in response to salinity. Quantities of transcription factors in the salt stress response gene of Y078 were found to be down-regulated, which may be a reason for the seed germination rate under salt stress. Meanwhile, some up-regulated transcription factors in these transcripts, such as AP2 domain class transcription factor (Unigene057115), heat shock protein (Unigene035187, Unigene028646, Unigene026005), MYB-like transcription factor family protein (Unigene037888) were also observed, indicating that these transcription factors may be key gene regulators of seed germination with salt stress in faba bean.

## Conclusion

In this study, some critical genes that may be correlated to salt tolerance of faba bean were identified between the salinity-tolerant and -sensitive faba bean varieties based on high-throughput RNA-Seq method. Genes involved in cell wall, chromatin remodeling, transcript or translation, transcriptional gene silencing, were significantly up-regulated, whereas genes involved in hormone metabolism of ABA and ethylene were down-regulated in the salt-tolerant variety with salt treatment. Meanwhile, the related transcription factors also play important roles in regulation of seed germination under salt stress. These results facilitate revealing the genetic mechanism of seed germination in response to salinity and will contribute to the further molecular breeding for salinity tolerant crops.

## Materials and methods

### Plant materials

Evaluation the tolerance of seeds to salt during germination was carried out as the protocol described by Chinese Crop Germplasm Resouce (http://www.cgrchina.cn/wp-content/uploads/2016/05/2-21-3.pdf) with minor modification. Twenty-five seeds were soaked in 8% sodium hypochlorite solution for 15 minutes, then rinsed with deionized water for 3 time, and placed in sterilized Petri dishes (12-cm diameter) with one sheet of filter paper. The dishes were added 15 mL NaCl solutions with the concentration of 200 mM or deionized water, and kept in a germination chamber in dark at 25 °C. Every 24 h, the seeds were taken out and rinsed with deionized water, then placed back into dishes with new filter paper. Another 15 mL NaCl solution (200 mM) or deionized water were newly added and kept in a germination chamber in dark at 25 °C again. The germinated seeds were counted every 24 h to calculate the germination rate based on the criteria that the radicle length was equivalent to the seed length and the cotyledons were intact or the damage is less than 1/3 of cotyledons, respectively. The seed germination experiment was conducted with three biological replicates for both the salt treatment and control groups. The salt tolerance was evaluated by the relative salt damage rate (RSDR), which was calculated in the formulation of RSDR = (GRc - GRt)/GRc. GRc represents the number of germinated seeds under control condition, and GRt is the number of germinated seeds under salt treatment. The salt tolerance can be ranked into five grades based on the RSDR, namely, high tolerance (RSDR < 20%), tolerance (20% ≤ RSDR < 40%), mediate tolerance (40% ≤ RSDR < 60%), sensitive (60% ≤ RSDR < 80%), high sensitive (RSDR ≥ 80%). For RNA-seq and qRT-PCR experiments, the 3/4 portion of the cotyledons were excised from the germinating seeds at 16 h or 24 h and discarded. The 1/4 portion of cotyledons with embryos were frozen in liquid nitrogen immediately, and kept at −80 °C refrigerator.

### Library construction and sequencing

Total RNA was isolated with RNA extract kit (Huayueyang, China), and Oligo (dT) beads were used to enrich mRNAby TruSeq RNA Sample Preparation Kit (Illumina, CA, USA). Then the enriched mRNA was fragmented shortly by fragmentation buffer and the first-stand cDNA was reverse-transcripted by random primers. Second-strand cDNA were then synthesized in buffer containing DNA polymerase I, RNase H, dNTP. After purification of cDNA fragments by Qia Quick PCR extraction kit, theends were repaired and the poly(A) were added. Then the Illumina sequencing adapters were ligated. The size of ligation products were -selected by agarose gel electrophoresis, then were used as templates for PCR amplified. The amplified products were sequenced on an Illumina Hiseq. 4000 using a PE125 flow cell by Sa gene Biotech Co. Ltd (Guangzhou, China).

### Quality control and *de novo* assembly

the filtering is further performed to gain highly qualitative clean reads based on the following principless:1) the reads with the adapter and the reads with more than 10% of unknown nucleotides (N) were removed; 2) the low-quality readings with Q value ≤ 10 bases were trimmed. The transcripts were assembled using Trinity program^34^ with Kmer = 25. The unigenes were annotated using BLASTX (http://www.ncbi.nlm.nih.gov/BLAST/) against Nr (the National Center for Biotechnology Information (NCBI) non-redundant protein database), Swiss-Prot (http://www.expasy.ch/sprot), KEGG (the Kyoto Encyclopedia of Genes and Genomes database), and COG/KOG (the Cluster of Orthologous Groups database) an a 1e-5 threshold of E-value. FPKM was used to normalize the gene expression abundance which is influenced by gene length and sequencing discrepancy due to calculation on gene expression^35^. The FPKM values of each unigene were used to calculate the pearson correlation coefficients among the 8 samples at each representative time point. The edgeR package was used to analyze the principal component for all 24 samples^36^.

### DEGs identifying and function categorizing

DEGs among the experimental groups was detected using the edgeR package in R. the statistical significance of expression difference was judged by the test, and the FDR served for *p*-value threshold in multiple testing. In current study, a level of FPKM ≥ 0.1, |log2 fold change | ≥1, and FDR ≤ 0.001 was set to identify DEGs in the comparison of each pairwise between phases. The GO annotations were assigned to the DEGs to further characterize their function by Blast2GO^37^. The pathway annotations were blasted against the KEGG database to assign the metabolic of DEGs. Hyper-geometric tests were performed to analyze GO and KEGG pathway enrichment with the whole seed transcriptome as the background.

### Validation with qRT-PCR assay

The total RNA of T1–16, T1–24, T2–16, and T2–24 were used for qRT-PCR experiment. qRT-PCR assay was conducted on the 24 total RNA samples. Each 5 μg of total RNA was used to synthesize the first-strand cDNA by the Super Script First-Strand cDNA Synthesis Kit (Invitrogen, USA). Primers for 24 selected genes were listed in Table S10. The reactions were conducted on the BIO-RAD CFX Connnect^TM^ Optics Module System (BIO-RAD, USA) according to the manufacturer’s instructions. The reaction was carried out in 10 μl volumes consist of 5 μl of SYBR Premix Ex Taq (Tiangan, China), 0.5 μl of each primer (10 μM), 2 μl of cDNA template, and 2 μl of RNase-free water. Three biological replicates and two technical replicates were conducted for each gene. The reaction procedure was as follows: 95 °C for 30 s; 40 cycles of 95 °C for 5 s and 60 °C for 30 s. The melting curve was analyzed to confirm the primer specificity. The 2^−ΔΔ^Ct method was adopt to calculate the relative expression levels of the tested genes. The housekeeping gene NADH dehydrogenase subunit 4 (NADHD4) was used as the internal reference gene as descripted by^38^.

## Supplementary information

Supplementary materials.

Table S5.

Table S6.

Table S7.

Table S8.

Table S9.

## Data Availability

The datasets generated during and/or analyzed during the current study are available in the NCBI Gene Bank database (https://submit.ncbi.nlm.nih.gov/subs/sra/). Accession code: PRJNA591424.
