# Supplementary material for: Transcriptome profile analysis of two Vicia faba cultivars with contrasting salinity tolerance during seed germination
Source: Sci Rep. 2020 Apr 29;10:7250. doi: 10.1038/s41598-020-64288-7 (PMC7190719; doi:10.1038/s41598-020-64288-7)
Supplement: Supplementary file 1 — Supplementary materials. [file 41598_2020_64288_MOESM1_ESM.pdf]

# Transcription profile analysis of two *Vicia faba* cultivars with contrasting salinity tolerance during seed germination

Fangwen Yang[1#], Hongwei Chen[2#], Changyan Liu[2], Li Li[2], Liangjun Liu[2], Xuesong Han[2], Zhenghuang Wan[2*], Aihua Sha[1*]

[1]Hubei Collaborative Innovation Center for Grain Industry, Yangtze University, Jingzhou, P.R. China
[2]Institute of Food Crops, Hubei Academy of Agricultural Sciences/Hubei Key Laboratory of Food Crop Germplasm and Genetic
[#]these authors contribute equally to this work
*Correspondence

**Supplementary Material**

**Supplementary Figure S1. Results of sequence saturation analysis**

**Supplementary Table S1. Statistics of sequencing reads of the *Vicia faba* seeds samples**

**Supplementary Table S2. Statistics of assembled unigenes and transcripts**

**Supplementary Table S3. Ratios of the clean reads from samples mapping to reference transcripts**

**Supplementary Table S4. Statistics of gene expression levels in different samples**

**Supplementary Table S10. Primer sequences used for real time qPCR**

**Figure S1. Results of sequence saturation analysis**

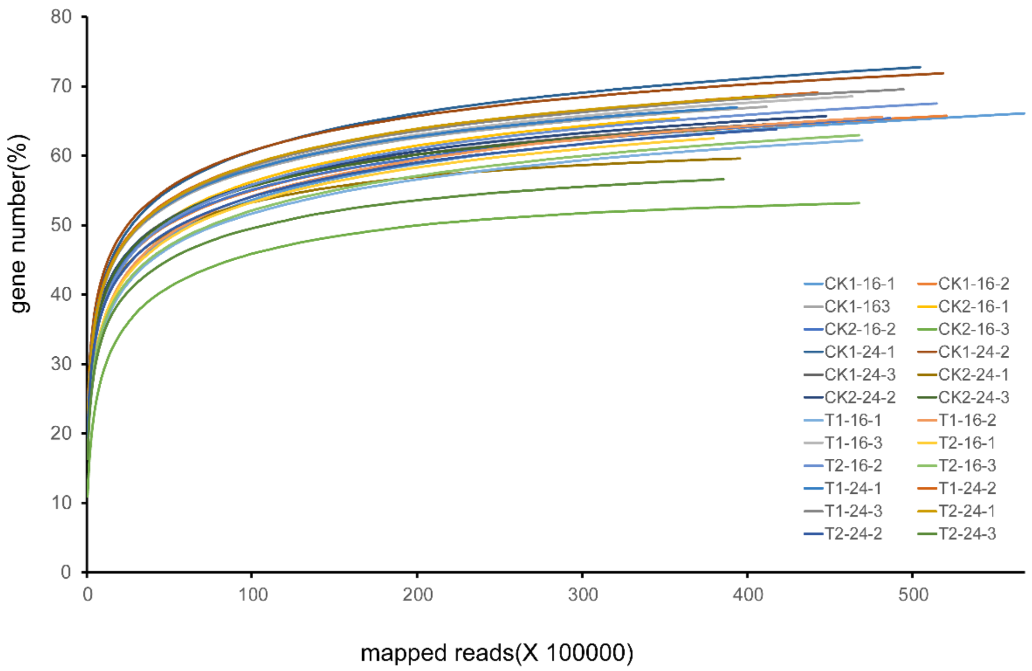

Table S1 Statistics of sequencing reads of *Vicia faba* seeds

| Samples | Raw | | | Clean | | |
|---|---|---|---|---|---|---|
| | Bases | Reads | Length (Max/Mean/min | Bases (%) | Reads (%) | Length (Max/Mean/min) |
| T1-16 | | | | | | |
| Bio Rep1 | 14307820599 | 95278858 | 151/150/35 | 14151067996(98.90%) | 94162558(98.83%) | 151/150/50 |
| Bio Rep2 | 14592765399 | 97229688 | 151/150/35 | 14474950112(99.19%) | 96411394(99.16%) | 151/150/50 |
| Bio Rep3 | 14047724467 | 93521838 | 151/150/35 | 13955732669(99.35%) | 92886898(99.32%) | 151/150/50 |
| CK1-16 | | | | | | |
| Bio Rep1 | 17175182097 | 114398882 | 151/150/35 | 17063051799(99.35%) | 113622522(99.32%) | 151/150/50 |
| Bio Rep2 | 15734292819 | 104755410 | 151/150/35 | 15660853028(99.53%) | 104256824(99.52%) | 151/150/50 |
| Bio Rep3 | 12529045935 | 83404580 | 151/150/35 | 12404190138(99.00%) | 82552738(98.98%) | 151/150/50 |
| T2-16 | | | | | | |
| Bio Rep1 | 11559892710 | 76983890 | 151/150/35 | 11435185131(98.92%) | 76128350(98.89%) | 151/150/50 |
| Bio Rep2 | 15585736874 | 103822458 | 151/150/35 | 15492758033(99.40%) | 103194698(99.40%) | 151/150/50 |
| Bio Rep3 | 14177045493 | 94386836 | 151/150/35 | 14067629806(99.23%) | 93636940(99.21%) | 151/150/50 |
| CK2-16 | | | | | | |
| Bio Rep1 | 10902330083 | 72600814 | 151/150/35 | 10794017852(99.01%) | 71859828(98.98%) | 151/150/50 |
| Bio Rep2 | 14705360654 | 97879282 | 151/150/35 | 14649328864(99.62%) | 97490884(99.60%) | 151/150/50 |
| Bio Rep3 | 14250195057 | 95298108 | 151/149/35 | 14034873181(98.49%) | 93627216(98.25%) | 151/149/50 |
| T1-24 | | | | | | |
| Bio Rep1 | 12001586555 | 80115690 | 151/149/35 | 11847949696(98.72%) | 78976524(98.58%) | 151/150/50 |
| Bio Rep2 | 13433400558 | 89452984 | 151/150/35 | 13333264413(99.25%) | 88768118(99.23%) | 151/150/50 |
| Bio Rep3 | 14974378048 | 99749908 | 151/150/35 | 14866734492(99.28%) | 99010034(99.26%) | 151/150/50 |
| CK1-24 | | | | | | |
| Bio Rep1 | 15278910818 | 101749094 | 151/150/35 | 15189494988(99.41%) | 101136174(99.40%) | 151/150/50 |
| Bio Rep2 | 15691327963 | 104546462 | 151/150/35 | 15596308386(99.39%) | 103892806(99.37%) | 151/150/50 |
| Bio Rep3 | 12725370377 | 84769094 | 151/149/35 | 12571541828(98.79%) | 83663116(98.70%) | 151/150/50 |
| T2-24 | | | | | | |
| Bio Rep1 | 12633457381 | 84210238 | 151/149/35 | 12540674263(99.27%) | 83558766(99.23%) | 151/150/50 |
| Bio Rep2 | 12711596559 | 84648324 | 151/150/35 | 12573788964(98.92%) | 83675048(98.85%) | 151/150/50 |
| Bio Rep3 | 11808371837 | 78878526 | 151/149/35 | 11623254742(98.43%) | 77382898(98.10%) | 151/150/50 |
| CK2-24 | | | | | | |
| Bio Rep1 | 12191435242 | 81774784 | 151/149/35 | 11917304763(97.75%) | 79384390(97.08%) | 151/150/50 |
| Bio Rep2 | 13678673344 | 91486038 | 151/149/35 | 13454585881(98.36%) | 89689156(98.04%) | 151/150/50 |
| Bio Rep3 | 11636640889 | 77865054 | 151/149/35 | 11436371745(98.28%) | 76203204(97.87%) | 151/150/50 |

T1-16, Y134 treat with sanity at 16 h; CK1-16, Y134 treated with water at 16 h; T2-16, Y078 treat with sanity at 16 h; CK2-16, Y078 treated with water at 16 h; T1-24: Y134 treat with sanity at 24 h; CK1-24, Y134 treated with water at 24 h; T2-24, Y078 treat with sanity at 16 h; CK2-24, Y078 treated with water at 24 h

Table S2 Statistics of assembled unigenes and transcripts

| Type | Amount | GC% | N50 (bp) | Mean Length (nt) | Min Length (nt) | Max Length (nt) | Assembled Bases (nt) |
|---|---|---|---|---|---|---|---|
| Unigenes | 116,093 | 38.3344 | 1599 | 850 | 201 | 19795 | 98772621 |
| Transcripts | 207,506 | 38.1107 | 1964 | 1163 | 201 | 19795 | 241496851 |

Table S3 Statistics of sequencing reads of samples mapping to reference genome

| Sample | Number of pair reads | Number of unique mapped reads | Number of multi mapped reads | Number of mapped reads | Mapping ratio |
|---|---|---|---|---|---|
| T1-16 | | | | | |
| Bio Rep1 | 47081279 | 12710631 | 15392234 | 28102865 | 59.69% |
| Bio Rep2 | 48205697 | 14980494 | 19045540 | 34026034 | 70.59% |
| Bio Rep3 | 46443449 | 12539119 | 21622721 | 34161840 | 73.56% |
| CK1-16 | | | | | |
| Bio Rep1 | 56811261 | 13949302 | 17324280 | 31273582 | 55.05% |
| Bio Rep2 | 52128412 | 15237628 | 29141265 | 44378893 | 85.13% |
| Bio Rep3 | 41276369 | 9815281 | 21727458 | 31542739 | 76.42% |
| T2-16 | | | | | |
| Bio Rep1 | 38064175 | 11253632 | 14714166 | 25967798 | 68.22% |
| Bio Rep2 | 51597349 | 13465300 | 17980243 | 31445543 | 60.94% |
| Bio Rep3 | 46818470 | 7152727 | 11984286 | 19137013 | 40.87% |
| CK2-16 | | | | | |
| Bio Rep1 | 35929914 | 12648457 | 15240517 | 27888974 | 77.62% |
| Bio Rep2 | 48745442 | 13037981 | 23589814 | 36627795 | 75.14% |
| Bio Rep3 | 46813608 | 10406377 | 9131813 | 19538190 | 41.74% |
| T1-24 | | | | | |
| Bio Rep1 | 39488262 | 11530057 | 18159484 | 29689541 | 75.19% |
| Bio Rep2 | 44384059 | 13200551 | 21972252 | 35172803 | 79.25% |
| Bio Rep3 | 49505017 | 14470932 | 25454663 | 39925595 | 80.65% |
| CK1-24 | | | | | |
| Bio Rep1 | 50568087 | 11748622 | 21416838 | 33165460 | 65.59% |
| Bio Rep2 | 51946403 | 13847656 | 28473844 | 42321500 | 81.47% |
| Bio Rep3 | 41831558 | 11916035 | 20288991 | 32205026 | 76.99% |
| T2-24 | | | | | |
| Bio Rep1 | 41779383 | 13380734 | 21087636 | 34468370 | 82.50% |
| Bio Rep2 | 41837524 | 12382862 | 16269688 | 28652550 | 68.49% |
| Bio Rep3 | 38691449 | 14368222 | 14420085 | 28788307 | 74.40% |
| CK2-24 | | | | | |
| Bio Rep1 | 39692195 | 13776860 | 16186053 | 29962913 | 75.49% |
| Bio Rep2 | 44844578 | 12783444 | 20616235 | 33399679 | 74.48% |
| Bio Rep3 | 38101602 | 11279841 | 18131233 | 29411074 | 77.19% |

T1-16, Y134 treated with salinity at 16 h; CK1-16, Y134 treated with water at 16 h; T2-16, Y078 treated with salinity at 16 h; CK2-16, Y078 treated with water at 16 h; T1-24, Y134 treated with salinity at 24 h; CK1-24, Y134 treated with water at 24 h; T2-24, Y078 treat with salinity at 16 h; CK2-24, Y078 treated with water at 24 h

Table S4 Statistics of expressed genes in different samples

| Samples | Ref_Count | Ref_Ratio | Samples | Ref_Count | Ref_Ratio |
|---|---|---|---|---|---|
| T1-16 | | | T2-16 | | |
| Bio-Rep1 | 47723 | 41.11% | Bio-Rep1 | 48471 | 41.75% |
| Bio-Rep2 | 51850 | 44.66% | Bio-Rep2 | 54925 | 47.31% |
| Bio-Rep3 | 56230 | 48.44% | Bio-Rep3 | 48698 | 41.95% |
| total | 69232 | 59.64% | total | 67756 | 58.37% |
| CK1-16 | | | CK2-16 | | |
| Bio-Rep1 | 53052 | 45.70% | Bio-Rep1 | 51730 | 44.56% |
| Bio-Rep2 | 51515 | 44.38% | Bio-Rep2 | 51629 | 44.47% |
| Bio-Rep3 | 53694 | 46.25% | Bio-Rep3 | 37581 | 32.37% |
| total | 71107 | 61.25% | total | 70026 | 60.32% |
| T1-24 | | | T2-24 | | |
| Bio-Rep1 | 52740 | 45.43% | Bio-Rep1 | 55633 | 47.92% |
| Bio-Rep2 | 56386 | 48.57% | Bio-Rep2 | 49730 | 42.84% |
| Bio-Rep3 | 57470 | 49.50% | Bio-Rep3 | 41112 | 35.41% |
| total | 73674 | 63.46% | total | 67644 | 58.27% |
| CK1-24 | | | CK2-24 | | |
| Bio-Rep1 | 62546 | 53.88% | Bio-Rep1 | 44225 | 38.10% |
| Bio-Rep2 | 60919 | 52.48% | Bio-Rep2 | 50430 | 43.44% |
| Bio-Rep3 | 50985 | 43.92% | Bio-Rep3 | 48492 | 41.77% |
| total | 77892 | 67.10% | total | 65502 | 56.42% |

Ref_count, the number of genes detected; Ref_Ratio, the percentage ratio of genes detected in each sample to all transcribed genes. T1-16, Y134 treat with salinity at 16 h; CK1-16, Y134 treated with water at 16 h; T2-16, Y078 treat with salinity at 16 h; CK2-16, Y078 treated with water at 16 h; T1-24: Y134 treat with salinity at 24 h; CK1-24, Y134 treated with water at 24 h; T2-24, Y078 treat with salinity at 16 h; CK2-24, Y078 treated with water at 24 h

Table S10 Primers for real time PCR

| GeneID | Left/right | primers (5’to 3’)Encoded protein |
|---|---|---|
| Unigene072411 | ACATCATAACTCGCACAGCA | alpha/beta fold hydrolase |
| | CTGCAACATCAGTGCCAAGT | |
| Unigene047182 | TGGTAGGATGTAAGCACTGAAGC | zinc ion-binding protein |
| | GAGAATGGAGATGTAGATCACGCT | |
| Unigene012043 | ACACCACCACCACGATCATC | late embryogenesis abundant protein |
| | CGGCGACTGAGGTTCTGAAG | |
| Unigene039110 | TCAGATTCACACTAGCCCTGT | glycoside hydrolase family 1 protein |
| | AGATGGTATACGGTGAGTGGG | |
| Unigene029331 | TAAAATCAGCGACGTGGCCA | late embryogenesis abundant protein |
| | TGCTATCTCTCTAGTGGCGCT | |
| Unigene056053 | TCCTCCTCCTTTGTTCTTGCC | late embryogenesis abundant protein |
| | GCCAAGGAGCAAGTCGACAT | |
| Unigene046631 | TGAACGTGCAGCTACCTTCT | PREDICTED: autophagy-related protein 3 |
| | CCCTCACTTGCCTGGAAAAC | |
| Unigene059340 | GAACCAGGCCATCGAGTTTC | 1-phosphatidylinositol-3-phosphate 5-kinase |
| | GCCACAACTCCATGCTTTCA | |
| Unigene077612 | AGACCATCGTTGTTTGTGTCG | calcium-binding EF-hand protein |
| | GCTACCATTGTCCACTTCCTTGA | |
| Unigene030495 | TGGCTGAGATACGAGACCCA | PREDICTED:ethylene-responsive transcription factor ERF109-like |
| | CCTGCAACCTCACTATGTTCCT | |
| Unigene043854 | ATCTCACGCATTTCACACTCATTG | late embryogenesis abundantD-likeprotein |
| | AGTTTCAGATGGTGTCGATGAGTG | |
| Unigene050339 | AGCGGGTTTGTGGATGAGAG | zinc finger constans-like protein |
| | CAAACACACAGTCCACGCG | |
| Unigene044769 | AAGAGGGGCCTATTTTGCGA | PREDICTED: autophagy-related protein 8C-like isoform X1 |
| | AGCCTTTACCCTGACTTGCT | |
| Unigene050814 | AGAAGTCGCTTGTTGTTGAAGAATG | PREDICTED: zinc finger BED domain-containing protein |
| | CTTCATCATCACCCACTACAGCT | |
| Unigene053156 | AGTGTTCCCAGCAAGCAACT | PREDICTED: zinc finger BED domain-containing protein |
| | TGCTTGGTAGTGTATGAATTGGGA | |
| Unigene056052 | ACACGTGGCTGCTGAGTAC | late embryogenesis abundant protein |
| | GCCAAGGAGCAAGTCGACAT | |
| Unigene058754 | TGTTAGAGGCGGTTAGGAGGT | nuclear transcription factor Y subunitC8 |
| | ACGACGAGGAGGATGAGTGA | |
| Unigene062764 | CAACCCAGAAAAGTCCATAGTTGC | F-box/RNI/FBD-like domain protein |
| | CCTGGTGTCATCATCCGTTCA | |
| Unigene077619 | GCGAATCGGTTACTCACAGTG | calcium-binding EF-hand protein |
| | TGAGTGTAGAAATTTTAGATGGGGC | |
| Unigene073153 | TGGTGCTGAGGATAAATGTGAAGA | replication factor-Acarboxy-terminal domain protein |
| | GAATCCGTGTGTTACCTCCATCA | |
| Unigene082835 | ATGCGTCTTCCTGGGAGTTG | late embryogenesis abundant D-like protein |
| | GAACAGCCCAAACGTCATGC | |
| Unigene046387 | GAGGGCCGAATCAAGTTACCA | PREDICTED: zinc finger BED domain-containing protein |
| | ACTGTGCGCTTGTAGTTGCT | |
| Unigene083605 | GAGAGACAGAGCGATTGGGC | zinc finger,C3HC4 type (RINGfinger) protein |
| | GGTCTATAACGCTCTCTTGTGGC | |
| Unigene086054 | ATCCCACACCTCCCCTCAAT | late embryogenesis abundantprotein,group6 |
| | GCTTCTTGTTGGATCTGGGGT | |
| NADHD4-F | AGGGTTAGTGAGCACCATGC | |
| NADHD4-R | ATAGCCAAAGGGAATACGCC | |
